# Experimental assessment of chloride and sulfate ions on the durability of CO_2_ carbonated backfill materials

**DOI:** 10.1371/journal.pone.0343496

**Published:** 2026-04-01

**Authors:** Ichhuy Ngo, Ruizhi Yang, Kunpeng Yu, Zhiyang Zhao, Kaidan Zheng, Zhishang Zhang, Chengkun Peng, Hemeng Zhang

**Affiliations:** 1 Key Laboratory of Xinjiang Coal Resources Green Mining (Xinjiang Institute of Engineering), Ministry of Education, Urumqi, China; 2 School of Mines, China University of Mining and Technology, Xuzhou, China; 3 Key Laboratory of Mine Thermodynamic Disasters and Control of Ministry of Education, Liaoning Technical University, Huludao, China; Jazan University College of Engineering, SAUDI ARABIA

## Abstract

The present study investigates the durability of CO_2_-carbonated coal-based solid waste backfill materials under the deterioration effects of chloride and sulfate ions. The primary objective is to evaluate the feasibility of environmentally favorable, CO_2_ negative backfilling materials for application in coal mining goaf stabilization. Carbonated samples at various curing stages were immersed in chloride and sulfate solutions, with tap water serving as the reference medium. Several key aspects were assessed, including dimensional alterations, mechanical property variation, phase evolution, and microstructural characteristics. Key phases were identified via XRD and SEM-EDS. The results indicate that the degree of deterioration followed the order: Cl^-^ > tap water> SO_4_^2-^. Compared with tap water, chloride exposure caused more severe degradation, whereas sulfate exposure partially mitigated the deterioration. Compressive strength decreased by 43.9% and 20.6% after immersion in chloride and sulfate solutions, respectively. The pronounced difference in damages is attributed to the high diffusivity of chloride ion with concentrations approximately 2.5 times higher than sulfate ions at a 5 mm depth, which not only consumed CaCO_3_ and C-A-S-H gel but also promoted the formation of Friedel’s salt and facilitated anion exchange reactions. In contrast, sulfate exposure led to the formation of ettringite crystals after leaching, which, when maintained below a certain concentration, acted as a filling agent that reduced microstructural porosity. This study provides a fundamental insights into the performance of CO_2_-carbonated backfill materials in chloride- and sulfate-rich environments, offering guidance for their practical application in sustainable coal mine backfilling.

## 1. Introduction

The rapid growth of the economy drives a huge demand for the mineral resources, coal exploitation is one of the vital resources for energy supplies in China [1]. The expansion of coal mining industry has risen to a plethora mining related issue, involving the surface solid waste accumulation such as mine tailing, waste rock, coal gangue and fly ash, along with the augmentation of subsurface mine goaf [2–5]. According to the China Coal Processing and Utilization Association (CCPUA), the cumulative stockpile of coal solid waste is approximately 4.5 billion tons with an annual increment of 795 million tons [6]. At the same time, the subsurface openings associated with coal mining activities could trigger various mining-related disasters such as overburden subsidence, rock bursts, aquifer water loss, and ecological issues that bring substantial hazards to both human safety and environmental integrity [7–10]. In light of these challenges, proper strategies and mitigation means should be developed.

In recent years, cemented paste backfill (CPB) has emerged as a highly effective and clean mining approach, solving the combination issues of coal mining goaf and surface solid waste accumulation [11]. This technique involves the mixtures of mine tailings with cementitious materials and water, then pumping into the working face of the mine [12]. As ordinary Portland cement (OPC) was the predominant binder, the prepared CPB is not economically viable and also contributes to carbon emissions [13]. In this regard, alkali-activated materials (AAMs) have gained more popularity and stand as a better choice. Jin et al. (2024) [14] prepared an alkali activated slurry using iron tailing, and found that inclusion of fly ash promoted the flowability and stability of the slurry. Liu et al. (2024) [15] effectively disposed phosphorus slag paste backfill material using various activators such as Na_2_SO_4_, Na_2_SiO_3_, Na_2_CO_3_ and NaOH. They concluded that NaOH possessed a better early hydration, but Na_2_SO_4_ provided sufficient later strength and enhanced denser hydration products and refined the pore structure characteristics. Yang et al. (2023) [16] studied the mechanical properties and long-term stability of modified magnesium slag cemented coal-based solid waste backfill material, and found that the uniaxial compressive strength, pore characteristics and microstructure were affected by fly ash addition and curing temperature. The effects were related to the hydration reaction and reaction products. Tang et al. (2024) [17] proposed a novel paste backfill material using anaolian sand, red mud and fly ash as primary raw materials. The paste slurry was found following the Herschel-Bulkley model, and the mechanical strength reached 6.4 MPa when hardened. Additionally, the materials meet the class III groundwater standards. Li et al. (2024) [18] used oil shale residue, steel slag, and soda residue to synthesize all solid waste low carbon cemented backfill material. The formulated materials meet the backfill performance requirement, where the compressive strength reached 5.97 MPa and the slump value was 205 mm. Furthermore, the developed materials supersede the cement-based materials with a CO_2_ emission reduction of 86.9%.

On top of CO_2_ emission reduction by waste materials replacement, scholars deepen the investigation on the active CO_2_ utilization in backfill materials. Wu et al. (2024) [19] utilized magnesium oxide in magnesium slag and adopt MgCl_2_ as calcium carbonate crystal modifier via wet carbonation method. The compressive strength was enhanced by 22% by introducing 0.1 M MgCl_2_, and the CO_2_ sequestration reached 20.33 g CO_2_ per 100 g magnesium slag. Chen et al. (2022) [20] addressed the ability of carbon uptake of cemented paste backfill under low CO2 concentration conditions. Using 1.5% concentration of CO_2_ was found to increase the carbonation rate by 4 times comparing to natural curing. Ngo et al. (2023) [21] used CO_2_ as a coactivation agent to enhance the setting and mechanical properties of the coal gangue fly ash backfill materials. CO_2_ was found to shorten the setting time of backfill slurry to up to 90% and strengthen the harden backfill body by up to 5 times. Li et al. (2023) [22] evaluated the direct aqueous carbonation of industrial solid waste for CO_2_ carbonation and concluded that solid waste with a high Ca content has better carbonation activity at a maximum rate of 544.6 g-CO_2_/kg. It was added that the particle size, reaction temperature, solid to liquid ratio and CO_2_ concentration should controlled below 75 μm, at 60 °C, 100g/L and 15%, respectively. As Li et al. (2023) [23] suggested, the carbonation rate and efficiency were in accordance with the CaO content of the materials. High CaO concentration induces an alkaline environment that promotes the pozzolanic reaction, thus generating a large amount of CaCO_3_.

With regard to long-term safe implementation of the backfill materials, the durability is one of the most important parameters to be considered. Sun et al. (2024) [24] recently studied the durability of biomass-coal mixed combustion ash geopolymer backfill. The mass loss was 0.93% and the compressive strength decrease was 33.2% after the erosion of the compound salt solution. Liu et al. (2020) [25] performed a case study on the effect of water on mechanical properties and permeability of backfill materials implemented in Taiping Coal Mine, China. The backfill materials were found to increase in weakening coefficient due to the slaking process, but the strength of the material was considerably stable due to the low hydraulic conductivity. Chen et al. (2023) [26] addressed the durability of the geopolymer backfill consisting of sodium silicate, fly ash, sand and water in wet-dry cycles. Under the attack of Na_2_SO_4_ and CaCl_2_ solution dry-wet cycles, the variation of hydraulic conductivity, mass and volume in contaminant solutions were observed. The hydraulic conductivity was found to increase by two orders of magnitude after first dry-wet cycle. It is noticeable that most of the studies focused on the durability and variation in the mechanical performance of solid waste backfill materials under different solution attack conditions. Very limited investigation has involved with the durability of CO_2_ carbonated backfill materials at post water deteriorations. It is actually a significant topic to be considered to extend the safe implementation of CO_2_ carbonated backfill materials. As mentioned above, the carbonated backfill materials are mainly filled with CaCO_3_ crystals, which is sensitive to acid, mild acid, or even water interactions. In our previous study [27], the durability of carbonated fly ash backfill materials was addressed considering cation solution attacks. However, anionic solution deteriorations were missing.

Therefore, the importance of this study lies in addressing the durability of CO_2_ carbonated backfill materials, which are proposed as a novel carbon-negative solution for mine goaf filling. While CO_2_ carbonation not only immobilizes greenhouse gases but also valorizes coal-based solid waste, the chemical durability of such materials under aggressive underground environments-particularly in the presence of chloride and sulfate ions-remains poorly understood. These ions are commonly present in mine water and can critically impair the mechanical integrity and service life of backfill materials, potentially limiting their large-scale application.

In this regard, this study aims to investigate the resistance and deterioration effects of CO_2_ carbonated backfill materials when exposed to chloride and sulfate-rich environment. Without other protective means, the deteriorations were determined at different curing time from 3 to 56 d to understand how ion-induced damage influences strength loss at various stages. Meanwhile, the penetration depths of chloride and sulfate ions, and mass loss and diameter changes were also considered. The test results were associated with solution analysis, phase transformations, and microstructural evolution to discover the underlying mechanisms. The study provides essential insights into the feasibility, environmental reliability, and practical deployment of CO_2_ carbonated backfill materials in underground mining operations.

## 2. Materials and methods

### 2.1. Materials

**Fig 1:**
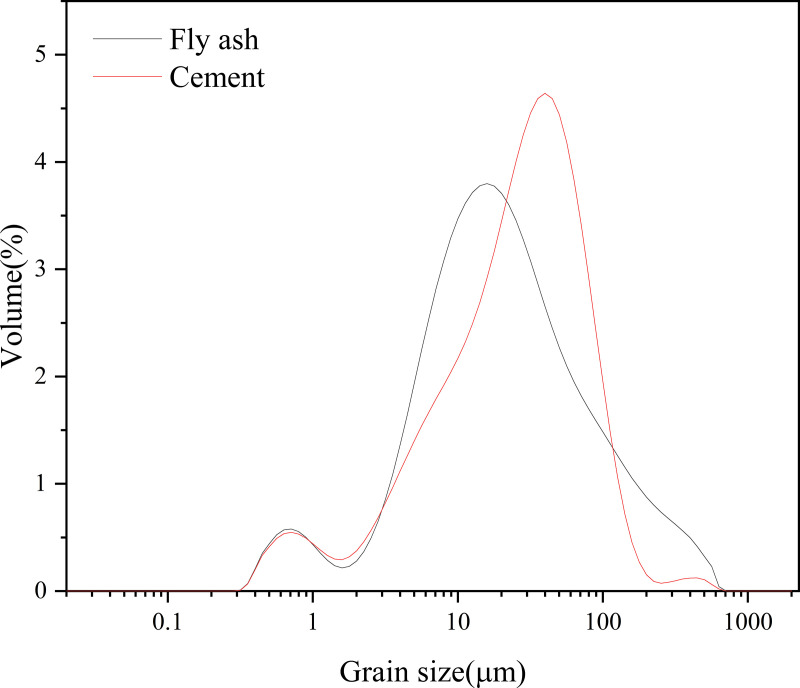
Grain size distribution of raw materials.

In this study, 80% of fly ash was used to replace the ordinary Portland cement (OPC). Fly ash was acquired from a coal-fired power plant in Zhengzhou, Henan Province, China. OPC PO 42.5 was purchased from Zhucheng Yangchun Co., Ltd. The collected fly ash was dried at 60 °C for 48 h to eliminate the residual water content. As the fly ash can pass the 200-mesh sieve, it was used as received. Both fly ash and cement were measured the cumulative particle size distribution using a laser diffraction method (Malvern model Mastersizer 2000, UK) as shown in Fig 1. The oxide and mineral composition of fly ash and cement were analyzed by X-ray fluorescence spectroscopy (XRF, Bruker model S8 Tiger spectrometer, Germany) and X-ray diffraction (XRD, Bruker model D8 Advance, Germany), respectively. The characterization results are shown in Table 1. The chemical properties indicated that fly ash mainly composed of SiO_2_ + Al_2_O_3_ + Fe_2_O_3_, and its sum is greater than 70%, and CaO is less than 18%. The used fly ash was then classified as Class F ash in accordance to the ASTM C618 standard [28]. The mineral compositions of fly ash are mullite, quartz and hematite, where the cements are tricalcium silicate (C_3_S), dicalcium (C_2_S), lime, and periclase.

**Table 1:** Oxide and mineral compositions of raw materials.

	Chemical composition (mass%)									
Sample	CaO	SiO_2_	Al_2_O_3_	Fe_2_O_3_	K_2_O	TiO_2_	MgO	SO_3_	P_2_O_3_	Other
Fly ash	2.04	51.85	32.14	4.09	1.14	1.09	0.75	0.67	0.21	6.02
Cement	48.76	25.73	7.98	3.54	1.02	0.60	5.01	4.12	0.24	3.00
	Mineral composition									
Fly ash	Mullite, quartz, hematite									
Cement	Tricalcium silicate, dicalcium silicate, lime and periclase									

The solid raw materials were coactivated using sodium silicate, which was purchased from Sigma-Aldrich (99% pure). The Na_2_O/SiO_2_ ratio of sodium silicate is 1.03 with a standard pH and viscosity of 11.5 and 5 mPa.s (10 wt% solution concentration), respectively at 25 °C. The CO_2_ gas used in the carbonation investigation was supplied by Xuzhou Luyou Gas Co., Ltd. Tap water was used to prepare the alkali solution and the deterioration water of Cl^-^ and SO_4_^2-^, where its initial pH is 6.8 ± 0.2. The deterioration water was synthesized in-house using NaCl and Na_2_SO_4_ supplied by Sigma Aldrich.

### 2.2. Preparation

**Fig 2:**
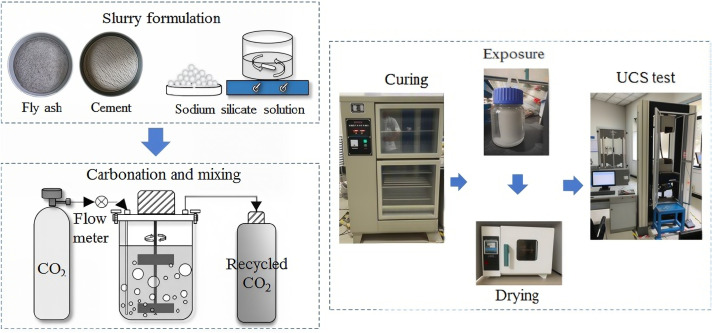
Overall workflow of the study.

The CO_2_ carbonated backfill samples were prepared in accordance to the Chinese standard (GB/T17671) [29]. To ensure a well-mixed backfill slurry, dry phase raw materials and activation solution were prepared separately, and the dosage of sodium silicate solution accounted for 30% by weight of the total mass of the mixture. Fly ash was added to cement in a 4:1 ratio and mechanically stirred at 500 rpm for 5 min. The alkali activation solution was formulated by adding 10 wt% of sodium silicate to tap water. The solution was stirred at 500 rpm for 30 min at room temperature. After which, the activator solution was mixed with the dry mixture in the reactor at 850 rpm for 3 min. Then the stirring speed was decreased to 500 rpm while CO_2_ gas was fed into the reactor at a constant rate of 1L/min for 20 min. It is worth highlighting that the dry to liquid ratio was selected based on our previous study [4]. The mortar was then molded into a cylindrical shape with a standard dimension of Φ 50 mm × 100 mm. The molds were covered to avoid water loss and allowed to stand for 48 h prior to demolding. The obtained samples were then transferred to a standard curing box with a constant temperature of 20 ± 2 °C and humidity of 95 ± 2%. The overall workflow of the this study is presented in Fig 2, and the details of the properties of the mixture are listed in Table 2.

**Table 2:** The mix ratio of CO_2_ carbonated backfill materials^a)^.

No.	F:C ratio	S:W ratio	Solid	Solution	CO_2_	Curing time
Sample	4:1	1:10	70wt%	30wt%	2,800 cm^3^	3/7/14/28/56d
	Diameter	Length	Volume	Mass	Density	UCS
Sample	4.92 cm	10.11 cm	192.2 cm^3^	313 g	1.63 g/cm^3^	5.88 MPa

a) F: fly ash, C: cement, S: sodium silicate, W: water.

### 2.3. Exposure condition

The immersion temperature was set at 25 ± 1°C, and the liquid-solid ratio was 10:1. A total of 3 cycles were performed using a saturation-drying cycle mode. The CO_2_ carbonated backfill material was subjected to seven different types exposures of deionized water, 2, 4, 6 wt% NaCl solution and 2, 4, 6 wt% Na_2_SO_4_ solution, as presented in Table 3. Backfill samples exposed to deionized water were presented as the reference group. At the predefined curing time of 3–56 days, the samples were exposed to deterioration solution for 7 days, followed by air dry at a constant temperature of 45 °C for 24 h.

**Table 3:** Synthesized NaCl and Na_2_SO_4_ deterioration water (wt%).

No.	NaCl	Na_2_SO_4_	pH
W0	0	0	6.8
C1	2		6.5
C2	4		6.1
C3	6		5.8
S1		2	6.7
S2		4	6.9
S3		6	7.0

#### 2.3.1. Chloride and sulfate concentration.

The concentrations of chloride and sulfate were measured by drilling the sample at a depth of 5, 10, 15, and 20 mm. The collected powder samples were then analyzed using Hitachi DR UV-3900 of ultra-violet and visible spectrometer. Prior testing, the powder samples were dissolved in deionized water and allowed to stand for 48 h.

#### 2.3.2. Solution analysis.

The leachate of the sample was collected after exposure for Al, Ca, Na, and Si analysis using Inductively Coupled Plasma Optical Emission Spectrometry (ICP-OES). The leachate sample was prepared by filtering 15 cm^3^ of sample through a 0.45 um filter and adding 1 cm^3^ of 7.85 mol/L nitric acid. Prior to analysis, the samples were refrigerated to reduce the chance that solids precipitated from the solution during storage.

#### 2.3.3. Mass and diameter change.

Prior to the mass and diameter change measurements, the dried samples were allowed to stand in the glass desiccator for at least 6 h. The mass change of deteriorated samples was weighted using an electronic scale with accuracy of 0.001 g. The measurements were repeated three times to ensure accuracy. The mass change was calculated following Eq. (1). For the same instance, the diameter variation of sample was monitored by an electric vernier caliper with an accuracy of 0.001 mm. The evaluation was as well repeated three times at the marked location to improve accuracy. The diameter change was calculated using Eq (2).


$$\Delta M_{i}=\frac{\left(M_{i}-M_{\text{0}}\right)}{M_{\text{0}}}\times 100\%$$ (1)

where $M_{\text{0}}$ is the initial mass $M_{i}$ is the mass after exposure.


$$\Delta D_{i}=\frac{\left(D_{i}-D_{\text{0}}\right)}{D_{\text{0}}}\times 100\%$$ (2)

where $D_{\text{0}}$ is the initial mass $D_{i}$ is the mass after exposure.

#### 2.3.4. Mini-slump and porosity test.

According to the standard GB/T 50080-2016, the Mini-slump test was carried out to evaluate the fluidity of fresh filling slurry. The test equipment adopted a truncated mini-slump cone with a top diameter of 50 mm, bottom diameter of 100 mm, and height of 150 mm. The porosity of the deteriorated backfill samples was determined by the water saturated method in accordance with the GB/T 34533-2023.Testing equipment includes an electronic balance (accuracy 0.01g) used for weighing dry mass and saturated mass, along with one YT-700 constant temperature drying oven.

#### 2.3.5. Uniaxial compressive strength test (UCS).

The samples were subjected to UCS test in accordance to the national standard (GB/T1761-2021) using a WDW-300 electronic universal testing machine. Displacement loading was controlled at a constant speed of 1 mm/min. The peak stress and displacement were recorded and utilized in further inspection. All tests were repeated three times.

#### 2.3.6. Morphological analysis.

The deteriorated samples by chloride and sulfate attacks were further analyzed to investigate the microstructure and the underlying deterioration mechanisms. A small portion of the sample collected during UCS test and its hydration was stopped with anhydrous ethanol. The sample was then oven dried at a constant temperature of 50 °C for 24 h. Scanning electron microscopy (SEM) and energy-dispersive X-ray spectroscopy (EDS) were performed using a field emission microscope (Hitachi model Regulus 8100) with an energy-dispersive spectrometer (EDXA model 560). The magnification was 100,000 times. The sample was observed in the 20-kV voltage field in a high vacuum condition. The spectrum from each measurement point was collected over a 30 s period. The working distance was 10.5 mm, and the spot was controlled at 3.5 mm.

#### 2.3.7. X-ray diffraction (XRD).

The mineral and chemical components analysis was performed using advanced X-ray diffractometer (Bruker model D8 Advance) with Cu Kα radiation (λ = 0.15419 nm) over 2θ ranging from 5 to 90^o^ and a step length of 0.02^o^. The deteriorated sample was ground in an agate mortar and sieved through a 200-mesh screen.

## 3. Results and discussion

### 3.1. Chloride and sulfate concentration

**Fig 3:**
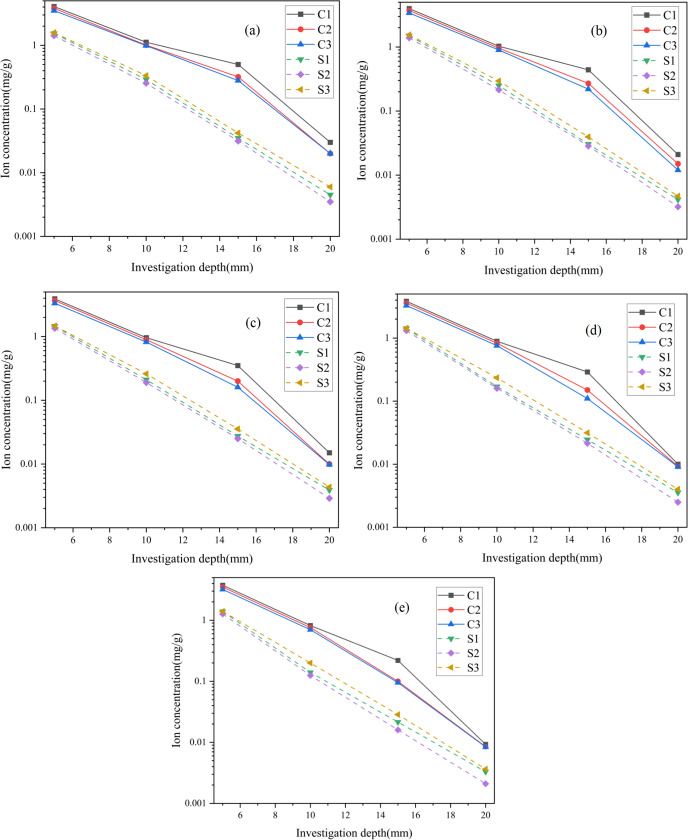
The concentration of chloride and sulfate ions (a) 3d, (b) 7d, (c) 14d, (d) 28d, and (e) 56d.

Fig 3 illustrates the penetration depth of chloride and sulfate ions within the samples. The ion concentration profiles for all exposures followed a similar trend, regardless of ion type: concentrations decreased with both penetration depth and curing time. At depth of 5 mm, the average concentrations of chloride and sulfate ions were 3.8 mg/g and 1.5 mg/g, respectively, which declined steadily to 1.04 mg/g and 0.29 mg/g at 10 mm. Beyond this depth, the reduction became less pronounced, reaching only 0.02 mg/g and 0.004 mg/g at 20 mm. As widely recognized, chloride and sulfate ions diffusion in cementitious materials is governed by concentration gradients, whereby ions migrate directionally from regions of high concentration at the material’s surface toward regions of lower concentration within the pore solution [30]. During this process, infiltrated ions are partly bonded or absorbed by the cementitious phases of the backfill matrix [31], limiting their continued penetration. Consequently, only a fraction of the ions advance deeper into the material, resulting in markedly lower concentrations at greater depths. These findings are consistent with the established diffusion mechanisms.

The concentration of chloride and sulfate ions decreased with increasing curing time. For instance, at depth of 5 mm, chloride and sulfate declined from averages of 3.8 mg/g and 1.5 mg/g to 3.48 mg/g and 1.35 mg/g, respectively, as the curing period was extended from 3 to 56 d. This reduction can be attributed to the enhanced formation of hydration products during prolonged curing, which provided additional binding sites that immobilized diffusing ions and restricted their further penetration. Notably, chloride ions exhibited a greater penetration capacity compared to sulfate ions. At equivalent depths, chloride concentrations were approximately 30% higher than those of sulfate. The lower detection of sulfate ions may be explained by the formation of ettringite near the surface and pore-matrix interface, which densified the microstructure and acted as a barrier to further sulfate ingress [32].

### 3.2. Solution analysis

**Fig 4:**
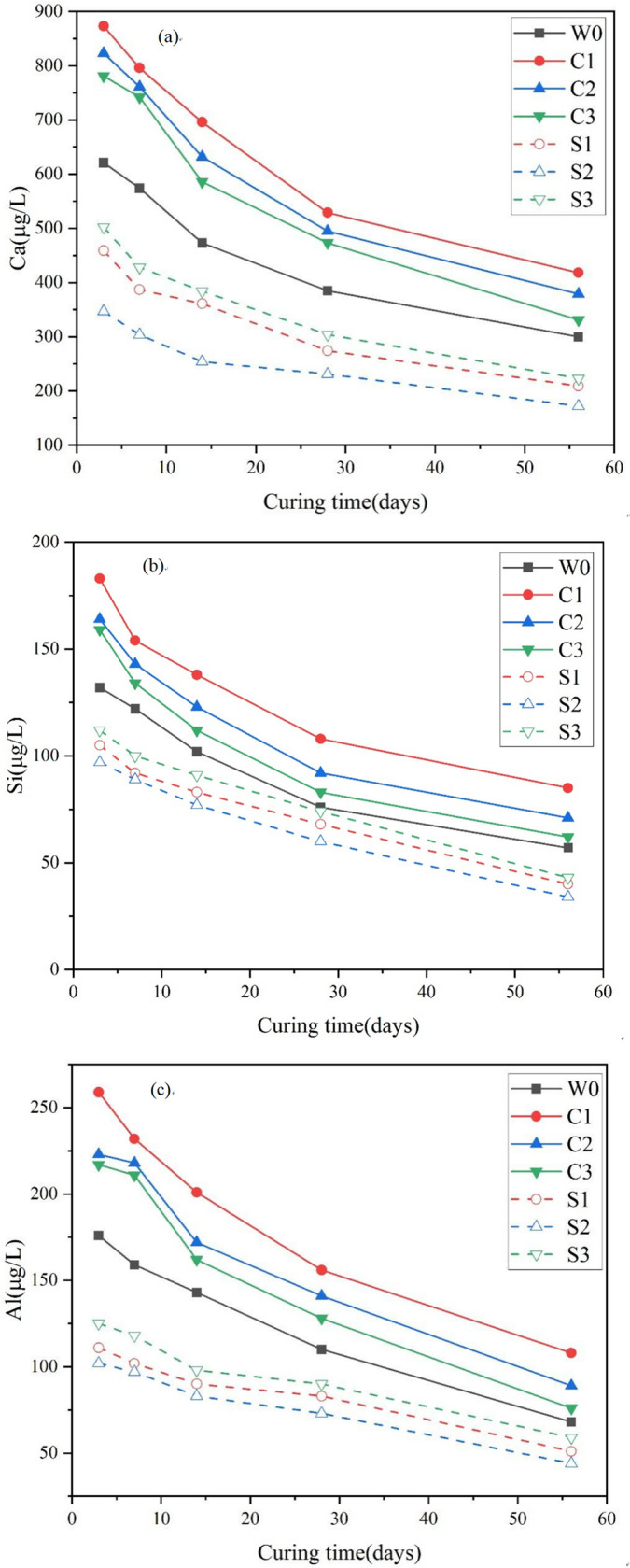
Leaching concentration within exposed solution (a) Ca ion, (b) Si ion, and (c) Al ion.

After exposure, the reacted solutions were collected and analyzed for their major ionic components, as shown in Fig 4.

Ca was the most abundantly released ion across all scenarios, followed by Al and Si, indicating that deterioration primarily affected by the hydration gel. The extent of ion release decreased with increasing curing time, reflecting improved stability of the backfill. For example, in the reference case (W0), Ca concentration decreased from 621 to 300 μg/L, Al from 176 to 68 μg/L, and Si from 132 to 57 μg/L as curing time extended from 3 to 56 d. This reduction is attributed to the greater development of hydration products during longer curing, which created a denser backfill matrix that limited solution ingress and subsequent ion leaching.

Using W0 as the baseline, it was further observed that ion release in chloride solutions (C1-C3) was consistently higher than in sulfate solution (S1-S3). For example, after 3 d of curing, Ca, Al, and Si concentrations in C1 were 873, 259, and 183 μg/L, respectively, compared with 459, 111, and 105 μg/L in S1. This behavior can be explained by the high transmissibility of chloride ions, which penetrate the backfill through diffusion, capillary wicking, and absorption [33]. During migration, a portion of chloride ions is bound within the cementitious matrix, while unbound ions remain in the pore solution [34]. These free chlorides participate in secondary reactions, altering the microstructure through the formation of Friedel’s salts, which occurs via adsorption and anion-exchange mechanisms [35,36]. The latter process facilitates calcium leaching from backfill body. In contrast, sulfate attack follows a “diffusion-reaction-expansion” or “diffusion-concentration-crystallization” mechanism. The sulfate reaction products, such as gypsum, ettringite, thaumasite, and magnesium silicate, continue to grow, resulting in significant volume expansion [37]. This expansion concentrates deterioration around capillary walls, leading to microcracking and accelerated erosion. The extent and nature of these corrosion products are strongly influenced by both pH and sulfate concentration [38]. Due to these mechanistic differences, the concentrations of leached ions in sulfate-exposed solutions were consistently lower than those in chloride-exposed solutions.

### 3.3. Mass and diameter changes

**Fig 5:**
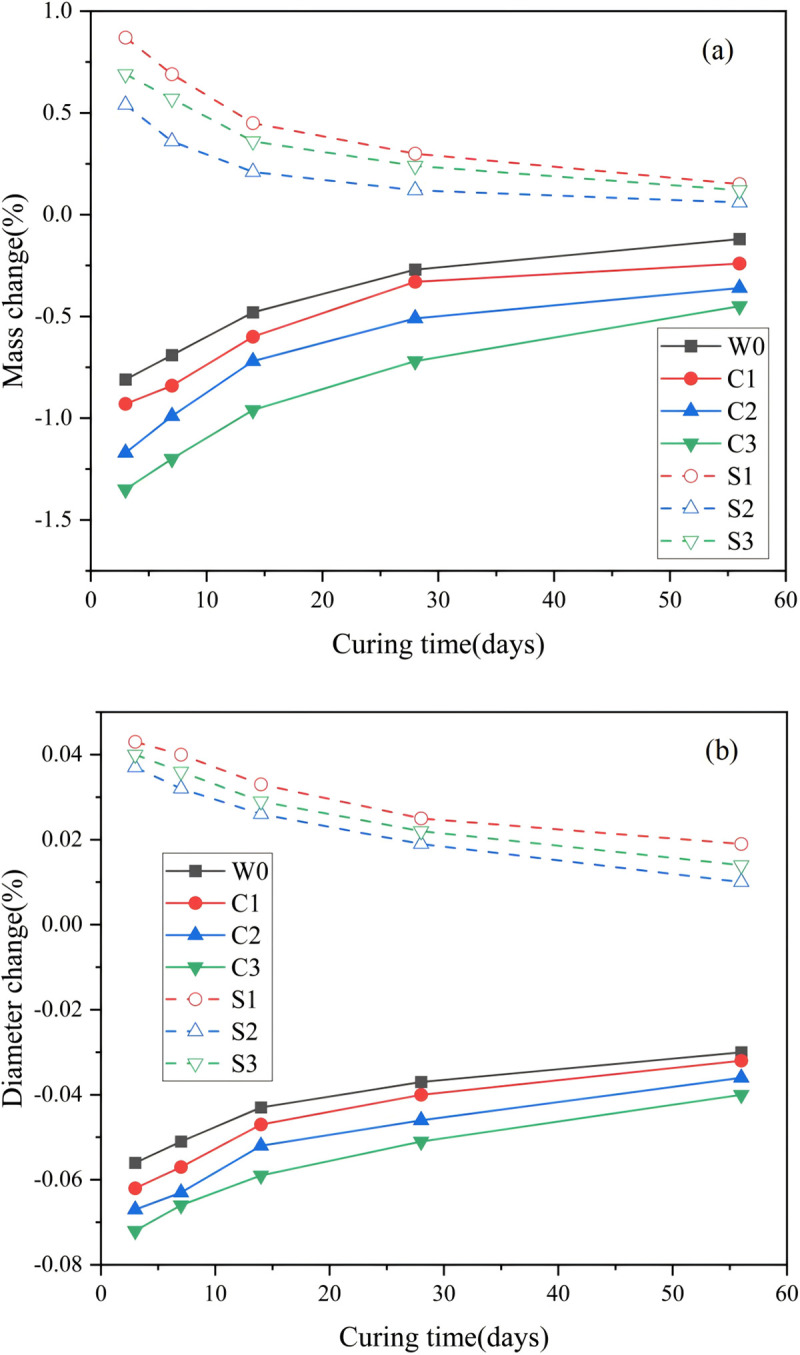
Evolution of (a) Mass and (b) diameter under different exposure conditions.

Fig 5 presents the mass and diameter changes of samples subjected to different curing times and exposure conditions.

The results show two distinct trends among the seven conditions tested. Samples exposed to deionized water and chloride solutions exhibited decreases in both mass and diameter, whereas those exposed to sulfate solutions displayed increases. For the control group (W0), mass decreased sharply between 3 and 14 d, followed by a gradual reduction up to 28 d, and then stabilized by 56 d. Changes in diameter followed a similar pattern. The reductions can be attributed to partial dissolution of surface and edge particles in water, as previously discussed [27]. The chloride-exposed group experienced more pronounced mass and diameter losses, likely due to internal interactions between Cl^-^ ions and hydration products. This interpretation is supported by solution analysis, which showed higher concentrations of leached Ca, Si, Al. In carbonated backfill materials, C-A-S-H hydrate forms initially but subsequently dissolves under chloride attack, as carbonate ions are replaced by chloride ions through an anion-exchange mechanism [39].

In contrast, sulfate exposed samples exhibited increases in both mass and diameter. This behavior reflects the fundamentally different deterioration mechanism compared with chloride attack. Sulfate diffusion initiates chemical reactions that generate expansive corrosion products, such as ettringite and gypsum, thereby increasing the overall volume [40]. However, this expansion effect diminished with longer curing times, as the denser backfill structure restricted the sulfate ingress. Additionally, the physical and adsorption of sulfate ions around Ca^2+^ sites may have induced repulsive forces within the matrix, further contributing to the observed dimensional changes [41].

### 3.4. Compressive strength

**Fig 6:**
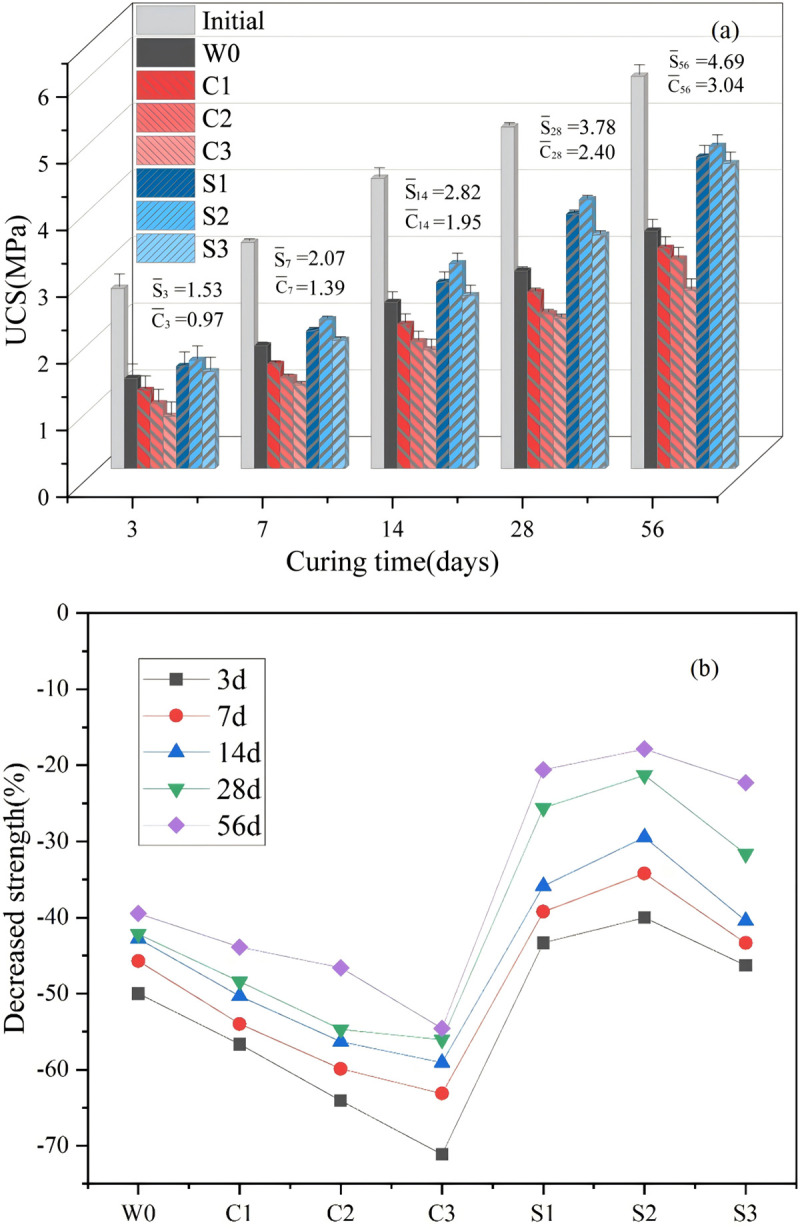
Influence of various exposure conditions on (a) compressive strength, (b) strength reduction.

Fig 6 shows the compressive strength of backfill materials exposed to different solutions under varying curing conditions.

Overall, the compressive strength of all carbonated samples decreased after exposure, regardless of the solution type. However, the degree of deterioration was influenced by curing duration and the concentration of dissolved ions. Longer curing times mitigated strength loss due to the enhanced formation of binding phases through the pozzolanic reaction of fly ash, the activation agent, and the precipitation of CaCO_3_ [4]. For instance, compressive strength declined from 2.7 to 1.35 MPa at 3 days and from 5.88 to 3.56 MPa at 56 days, corresponding to reductions of 50% and 39.5%, respectively.

Chloride exposure caused more severe deterioration compared to W0. In C1, compressive strength fell to 1.17 MPa at 3 days and 3.3 MPa at 56 days, representing reductions of 56.7% and 43.9%. The effect was amplified at higher chloride concentrations: C2 exhibited reductions of 64.1% and 46.6%, while C3 showed the most pronounced deterioration, with reductions of 71.1% at 3 days and 54.6% at 56 days. These results indicate that chloride-induced strength loss is highly dependent on ion concentration. Chloride permeation promotes the formation of Friedel’s salt through adsorption and anion-exchange, progressively altering the internal microstructure. Higher chloride concentrations increased ion diffusivity, thereby accelerating chemical reactions and structural degradation, consistent with previous findings [42].

In contrast, sulfate exposure induced less strength reduction compared with both W0 and chloride attack. For S1, compressive strength was 1.53 MPa at 3 days and 4.67 MPa at 56 days, corresponding to reductions of 43.3% and 20.6%. The reductions for S2 were 40% at 3 days and 17.9% at 56 days, while S3 samples exhibited 46.3% and 22.3% reductions, respectively. Unlike chloride attack, the deterioration trend under sulfate solutions showed a non-linear pattern: strength loss decreased from S1 to S2, then increased again at S3. This behavior is attributed to the swelling effect of corrosion products. Sulfate ions reacted with cementitious phases to form gypsum and ettringite, initially filling pores and densifying the microstructure at moderate concentrations (S2). However, at higher concentration (S3), excessive expansion exceeded the accommodation capacity of internal voids, inducing cracking and renewed strength loss [43].

### 3.5. Morphological analysis

**Fig 7:**
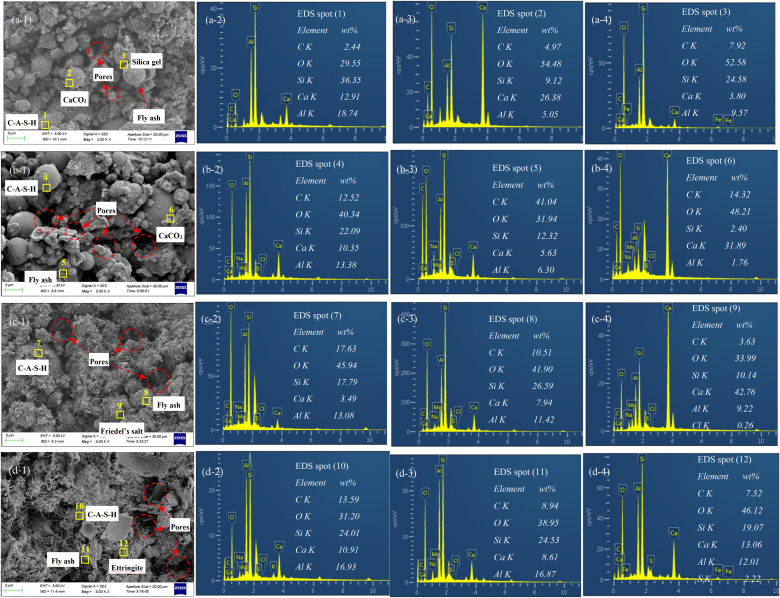
SEM images and representative dispersive spectroscopy (EDS) spectra of carbonated backfill samples after various exposures.

Fig 7 presents the microstructural features and corrosion product morphologies of carbonated backfill materials under different exposure conditions.

As shown in Fig 7a-1, the unexposed sample exhibited a dense structure in which particles were coated with flocculent phases. EDS analysis of spot 1 (Fig 7a-2) revealed high concentrations of Ca, Al, Si, and O, confirming the presence of C–A–S–H gel [44]. In addition, numerous white crystalline deposits were observed on the flocculent structures. These crystals (spot 2, Fig 7a-3) were rich in C, Ca, and O, characteristic of CaCO_3_, while the associated gelling agent (spot 3, Fig 7a-4) was composed mainly of Si and O, consistent with silica gel [44]. The combined presence of C–A–S–H gel, CaCO_3_, and silica gel contributed to the compact structure and strength of the carbonated backfill material. After exposure to deionized water (W0), the microstructure became noticeably looser with extensive pore development (Fig 7b-1). EDS spectra from spots 4–6 (Fig 7b-2 to 7b-4) indicated no new crystalline products; instead, leaching of Ca, Si, and Al occurred, consistent with solution analysis results. Strength reduction in this case was mainly attributed to dissolution of the binding phases (C–A–S–H and silica gel) and the loss of CaCO_3_ as a filling component.

Exposure to chloride solution induced more severe alterations (Fig 7c). C–A–S–H gels were swollen and degraded, reducing their binding capacity. EDS analysis of spot 7 (Fig 7c-2) showed substantial decreases in Ca, Al, and Si contents, while intact fly ash particles with clean surfaces were visible (spot 8), indicating weakened particle bonding and reduced structural integrity [4]. Additionally, flake-like crystals were observed (spot 9), which EDS confirmed as Friedel’s salt (high Ca, O, Al, and Cl; Fig 7c-4) [45]. These findings align with the strength reduction results: chloride ions penetrated the matrix, adsorbing Ca and Al through anion exchange, forming Friedel’s salt, and disrupting the stability of C–A–S–H and CaCO_3_.

**Fig 8:**
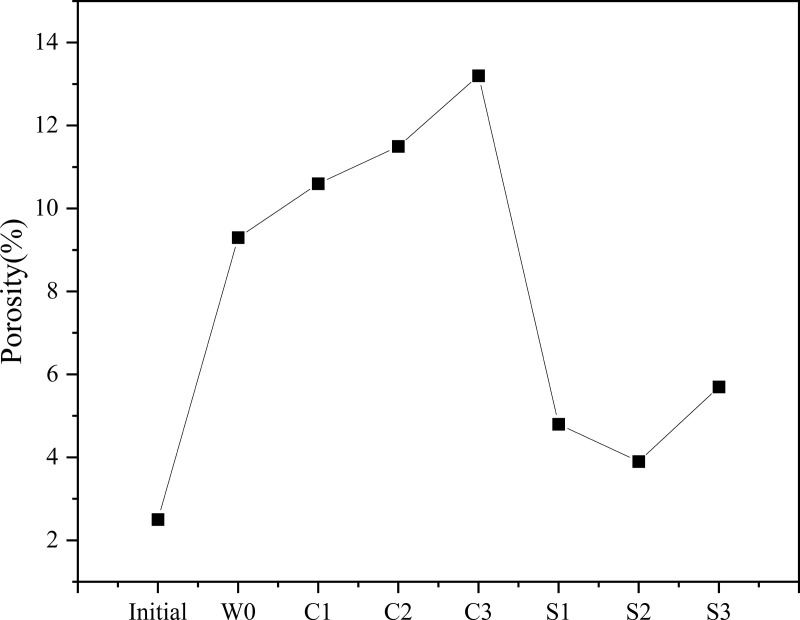
Porosity of backfill materials after exposure to various solutions.

In the case of sulfate exposure, the microstructure expanded and became irregularly porous (Fig 7d-1). Similar to chloride attack, C–A–S–H phases leached and lost binding ability, as evidenced at spot 10 (Fig 7d-2). Clean fly ash particle surfaces (spot 11, Fig 7d-3) further indicated poor bonding within the matrix. Importantly, sulfate reactions generated acicular crystals identified as ettringite (spot 12, Fig 7d-4), confirmed by EDS spectra showing high Ca, Al, O, and S concentrations [46]. The growth of ettringite induced internal expansion, which explains the observed increases in sample diameter after sulfate exposure. This expansive pressure caused microcracking and structural damage, consistent with the measured increases in porosity shown in Fig 8, ultimately leading to strength reduction.

### 3.6. Evolution of hydration and carbonation products

**Fig 9:**
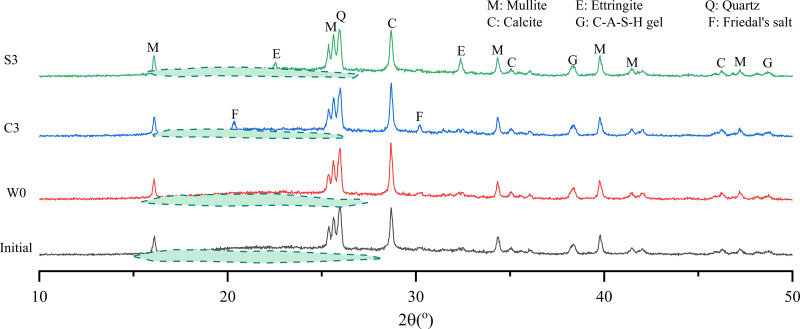
XRD diffraction peaks of carbonate backfill materials after exposure to various solutions.

Fig 9 presents the XRD diffraction patterns of carbonated backfill samples at the initial stage and after 56 days of exposure to different deterioration solutions.

In the initial state, the carbonated backfill consisted primarily of fly ash crystals, identified as mullite and calcite, along with an amorphous phase attributed to C–A–S–H gel, which appeared between 15o and 35o. As confirmed by SEM–EDS analysis, C–A–S–H gel served as the main binding agent, integrating fly ash particles and calcite crystals to enhance material strength. After exposure to water, chloride, and sulfate solutions, this amorphous phase was significantly reduced, indicating the loss of binding capability—consistent with the observed strength reduction and microstructural deterioration.

According to literature [47], the corrosion mechanism of cementitious materials under sulfate attack involves the dissolution of portlandite (Ca(OH)2), which reacts with incoming sulfate ions to form gypsum (Eq. 3). Calcium aluminate phases subsequently react to form monosulfoaluminate (AFm) or ettringite (AFt), while AFm may further convert into the more stable ettringite in the presence of gypsum (Eq. 4). Ettringite growth is expansive, generating internal stresses that induce microcracking and structural damage. In this study, strong diffraction peaks of ettringite were observed in the sulfate exposure group (S3), whereas samples immersed in tap water (W0) exhibited only weak or trace ettringite peaks. This confirms that new ettringite formed within the matrix during sulfate attack, consistent with the swelling and expansion observed in micromorphological analysis.


$$Ca(OH{)}_{\text{2}}+Na_{\text{2}}SO_{\text{4}}+2H_{\text{2}}O\to CaSO_{\text{4}}\cdot 2H_{\text{2}}O(Gypsum)+2NaOH$$ (3)

$$\begin{array}{c} 3CaO\cdot Al_{\text{2}}O_{\text{3}}+3(CaSO_{\text{4}}\cdot 2H_{\text{2}}O)+26H_{\text{2}}O\\ \to 3CaO\cdot Al_{\text{2}}O_{\text{3}}+3CaSO_{\text{4}}\cdot 32H_{\text{2}}O\left(Ettringite\right) \end{array}$$

$$\begin{array}{c} 2(CaSO_{\text{4}}\cdot 2H_{\text{2}}O)+3CaO\cdot Al_{\text{2}}O_{\text{3}}\cdot CaSO_{\text{4}}\cdot 12H_{\text{2}}O\left(AFm\right)+16H_{\text{2}}O\\ \to 3CaO\cdot Al_{\text{2}}O_{\text{3}}\cdot 3CaSO_{\text{4}}\cdot 32H_{\text{2}}O\left(Ettringite\right) \end{array}$$ (4)

For chloride exposure (C3), the XRD patterns revealed the disappearance of ettringite peaks and the emergence of distinct peaks corresponding to Friedel’s salt (3CaO·Al_2_O_3_·CaCl_2_·10H_2_O). This observation aligns with SEM–EDS results and supports the mechanism described in literature [48], wherein chloride ions, with a diffusion coefficient nearly twice that of sulfate, rapidly penetrate the backfill matrix and react with hydrated aluminate phases to form Friedel’s salt (Eq. 5). Moreover, chloride solutions were slightly acidic (pH 5.8–6.5, Table 3), which further exacerbated deterioration by promoting dissolution of binding phases and generating additional pathways that facilitated ion migration.


$$2Cl^{-}+Ca(OH{)}_{\text{2}}\to CaCl_{\text{2}}+2OH^{-}$$

$$\begin{array}{cc} & 2NaCl+3CaO\cdot Al_{\text{2}}O_{\text{3}}\cdot 6H_{\text{2}}O+CaCl_{\text{2}}+4H_{\text{2}}O\to 3CaO\cdot Al_{\text{2}}O_{\text{3}}\cdot CaCl_{\text{2}}\cdot 10H_{\text{2}}O+2NaOH\text{\textbackslash{}hspace\{0.17em}\}CaCl_{\text{2}}\\ & \;\;+3CaO\cdot Al_{\text{2}}O_{\text{3}}\cdot 6H_{\text{2}}O+CaCl_{\text{2}}+4H_{\text{2}}O\to 3CaO\cdot Al_{\text{2}}O_{\text{3}}\cdot CaCl_{\text{2}}\cdot 10H_{\text{2}}O \end{array}$$ (5)

### 3.7. Strengths and suggestions

This study evaluated the deterioration behavior of carbonated backfill materials after exposure to tap water, chloride, and sulfate solutions. The degree of strength reduction followed the order: Cl^-^ > tap water> SO_4_^2-^. Chloride exposure exerted the most severe influence, with compressive strength reductions exceeding 50% at early ages and remaining above 40% even after prolonged curing. This accelerated deterioration was attributed to the formation of Friedel’s salt and enhanced permeability, which facilitated internal chemical reactions and progressive weakening. In chloride-rich mine water environments, such degradation could substantially compromise the load-bearing capacity of backfill, threatening the long-term stability of goaf structures.

In contrast, sulfate attack resulted in comparatively moderate strength losses (<25% at 56 days) but progressed via a swelling–cracking mechanism. At moderate concentrations, expansive products such as gypsum and ettringite partially densified the matrix, temporarily mitigating deterioration. However, at higher concentrations, excessive expansion exceeded the pore volume, leading to microcracking and renewed strength loss. These findings highlight that chloride ions represent an immediate and sustained risk to structural integrity, whereas sulfate ions pose a time-dependent risk that may culminate in delayed failure. Accordingly, the development of ion-resistant mix designs and optimized curing strategies is essential to ensure the durability and stability of CO_2_-carbonated backfill materials in aggressive mine water environments.

### 3.8. Effect of saturation-drying cycle on material degradation

The sample were soaked for 7 days and then dried at 45 °C for 24 hours. After repeated three cycles, the mass of the sample increased by 3.2% compared with the initial value. The water absorption and dehydration in the process of saturation-drying cycle produce capillary adsorption effect in the sample, which makes the penetration rate of chloride ion and sulfate ion increase by 40% compared with tap water immersion. This significantly accelerates the migration of ions into the matrix.

This enhanced ion penetration further aggravates material degradation. In the chloride ion erosion group, the saturation-drying cycle increased the yield of Friedel ‘s salt in the specimen by 28% compared with tap water immersion. The dissolution of C-A-S-H gel was accelerated. The porosity of the sample increased by 12% compared with the initial state. After 56 days of curing, the compressive strength loss of the sample reached 54.2%. Under the erosion of medium concentration (4wt %) sulfate ions, the pore filling effect of ettringite partially offsets the deterioration of the material. The strength loss of the sample was 25.3%. The strength value was 20.6% higher than that of tap water immersion. However, under the erosion of high concentration (6wt %) sulfate ions, the saturation-drying cycle caused excessive expansion and cracking. The strength loss of the sample increased to 31.7%.

### 3.9. Fluidity and water content

The test results show that the slump of the slurry reaches 345 mm when the optimized mix ratio (the mass ratio of solid phase (cement + fly ash) to sodium silicate solution is 7:3) is adopted. The value exceeds the 200 mm required for the filling material. It ensures uniform flow in complex goaf, no segregation phenomenon, and no additional vibration. This mobility ensures transport efficiency and stability. The pumping pressure and the risk of blockage are reduced. At the same time, the bleeding amount of the material and the precipitation of the aggregate are reduced. The liquid content of wt 30% can fully promote the pozzolanic reaction, CO2 carbonization and alkali activation reaction, and the generated C-A-S-H gel and calcium carbonate crystal can form a dense matrix [49,50].

### 3.10. Discussion

The long-term in-situ stability of goaf is very important for coal mine safety. Chloride ions in the specimens cause continuous deterioration by generating Friedel ‘s salt and consuming key binding phases. In the harsh environment may damage the bearing capacity of the filling body. The formation of ettringite by sulfate ions at a medium concentration temporarily improves the compactness of the specimen. Long-term intrusion leads to excessive expansion and strength loss of the specimen. The stability of the specimen under mild conditions is better. A variety of factors in the complex in-situ environment may aggravate the deterioration beyond the scope of laboratory simulation.

The feasible strategies to improve durability mainly include the following points. Respectively, adding highly active mineral admixtures to refine the pore structure, applying protective coatings to achieve physical isolation, optimizing carbonization parameters to increase calcium carbonate content, and using anti-sulfate admixtures to regulate the formation of ettringite. At the environmental level, materials can realize the resource utilization of coal-based solid waste. The amount of CO2 released in the material is extremely low, and it can immobilize heavy metals without leaching of toxic ions, which is in line with the sustainable development goals. Future research should focus on on-site long-term monitoring and multi-ion synergistic erosion tests to further optimize materials to adapt to complex underground environments.

## 4. Conclusions

In this study, the durability and mechanical behavior of CO_2_-carbonated backfill materials were investigated under exposure to different deterioration solutions over curing periods ranging from 3 to 56 days.The experiments demonstrated that, regardless of the exposure solution, the mechanical properties and overall durability of the backfill improved with extended curing time. Considering the influence of dissolved ions, the degree of deterioration followed the order: Cl^-^ > tap water> SO_4_^2-^. Notably, positive correlations were observed between chloride ion penetration, mass loss, and diameter changes, which all increased with chloride concentration. In contrast, these parameters exhibited a non-linear trend under sulfate exposure, initially decreasing and then increasing with higher sulfate concentrations.

Microstructural observations provided direct evidence of the chemical interactions underlying deterioration. Chloride attack consumed CaCO_3_ and C–A–S–H gel while generating Friedel’s salt via anion exchange within micropores, leading to increased porosity and microcracking. Sulfate attack promoted the formation of ettringite, which initially reduced microvoids and partially densified the matrix at moderate concentrations. However, when ettringite formation exceeded a critical threshold, porosity increased, resulting in microcracks and further structural weakening. These findings elucidate the distinct mechanisms by which chloride and sulfate ions influence the durability of CO_2_-carbonated backfill materials.
